# Current trends in graft choice for primary anterior cruciate ligament reconstruction – part II: In-vivo kinematics, patient reported outcomes, re-rupture rates, strength recovery, return to sports and complications

**DOI:** 10.1186/s40634-023-00601-3

**Published:** 2023-04-04

**Authors:** Armin Runer, Laura Keeling, Nyaluma Wagala, Hans Nugraha, Emre Anil Özbek, Jonathan D. Hughes, Volker Musahl

**Affiliations:** 1 Department of Orthopaedic Surgery, UPMC Freddie Fu Sports Medicine Center, University of Pittsburgh, Pittsburgh, PA USA; 2 Department for Sports Orthopaedics, Klinikum Rechts der Isar, Technical University of Munich, Munich, Germany; 3 Department of Orthopaedic and Traumatology, Faculty of Medicine, University of Udayana, / Prof. Dr. I.G.N.G. Ngoerah General Hospital, Denpasar, Bali, Indonesia; 4 Department of Orthopedics and Traumatology, Ankara University, Ankara, Turkey; 5 Department of Orthopaedics, Institute of Clinical Sciences, Sahlgrenska Academy, University of Gothenburg, Gothenburg, Sweden

## Abstract

Postoperative patient satisfaction after anterior cruciate ligament reconstruction (ACL-R) is influenced mainly by the degree of pain, the need for reoperation, and functional performance in daily activities and sports. Graft choice has shown to have an influence on postoperative outcomes after ACL-R. While patient reported outcomes measurements do not differ between graft options, evidence shows that normal knee kinematics is not fully restored after ACL-R with an increase in postoperative anterior tibial translation (ATT). Postoperative graft rupture rates seem to favor bone-patella-tendon-bone (BPTB) and quadriceps tendon (QT) autografts over HT or allografts. While return to sports rates seem comparable between different graft types, postoperative extensor strength is reduced in patients with BPTB and QT whereas flexion strength is weakened in patients with HT. Postoperative donor site morbidity is highest in BPTB but comparable between HT and QT. With all graft options having advantages and drawbacks, graft choice must be individualized and chosen in accordance with the patient.

## Introduction

Pain, graft survival, and functional performance during daily activity and sport all significantly affect patient satisfaction following anterior cruciate ligament (ACL) reconstruction (ACL-R). Details about anatomy, biomechanics, graft fixation and incorporation commonly used autograft and allografts are reviewed in part I of this current concept paper. The following review will further highlight in-vivo analyses, patient reported outcomes (PROs), re-rupture rates, flexion and extension strength recovery, return to sport, and complications of the quadriceps tendon (QT), bone-patella-tendon-bone (BPTB) and hamstring tendon (HT) autograft as well as allografts. Unless otherwise specified, for the purposes of uniform comparison only studies using anteromedial portal drilling technique were included, as clinical and functional outcomes may differ with more traditional techniques [16].

### In-vivo analyses

Measuring in-vivo knee kinematics during daily and athletic activities is essential to detect abnormal joint mechanics and microinstability which may not present during routine clinical testing, yet may lead to accelerated joint degeneration [4].

ACL-R has been shown to have a significant impact on knee kinematics, with reconstructed knees more externally rotated and less flexed than the contralateral limb in the early stance phase of the running cycle one year postoperatively [13, 44, 121, 122]. Additionally, graft length was found to be 4 – 6 mm shorter compared to the native ACL at 6 and 24 months postoperatively throughout early stance [122]. While the clinical influence has yet to be determined, it can be hypothesized that a shorter and stiffer graft results in a more externally rotated tibia due to the oblique ACL fiber direction. This in turn may lead to an over-constrained joint in the early postoperative period [122]. However, over time there is an apparent decrease in external tibial rotation paired with graft lengthening and an increase in anterior tibial translation (ATT), indicating a stretching and functional remodeling of the graft [122].

Overall, the effect of different graft types on in-vivo kinematics remains inconclusive. For HT ACL-R an increased ATT during activity was reported and linked to a reduction in hamstring force [55]. Similarly, evidence shows that normal knee kinematics does not fully reestablish under weightbearing conditions after BPTB ACL-R even though anterior knee laxity measurements were restored during KT-1000 arthrometer testing [97]. A comparative study of HT- and BPTB ACL-R using dynamic biplanar radiography revealed no statistically significant difference in postoperative ATT between both graft options [54]. However, although not statistically significant, a higher ATT was measured in the HT group compared with BPTB during walking at 6 weeks. This again may be attributed to less posterior hamstring pull on the tibia in the early postoperative phase, which resolves after physical therapy and strength restoration [54].

### Patient reported outcome measures

Postoperative patient satisfaction is undoubtedly the most important outcome when it comes to ACL-R. While there is an abundance of short-, mid- and long-term literature comparing BPTB and HT, little is known about postoperative outcomes of QT. Although BPTB autograft has long been the gold standard in ACL–R, QT is gaining in popularity, especially among patients injured in pivoting sports and in those with concomitant medial collateral ligament injuries [7, 108].

To date, only two randomized controlled trials (RCTs) have compared clinical outcomes of BPTB and QT. Randomizing 51 patients using a transtibial ACL-R technique revealed no statistically significant difference in any of the reported PROs at two years postoperative [73]. Similar, no long-term differences were observed between quadriceps-tendon–patella bone autograft or BPTB in 60 athletes (Tegner > 6). In contrast, a multicenter, observational study reported significantly higher Lysholm scores for QT when compared to BPBT, yet similar results when compared to HT [92]. Several cohort studies as well as recent systematic reviews and meta-analyses support the findings of these randomized trials, demonstrating no significant difference in PROs between patients treated with QT or BPTB [21, 62, 86, 91, 100].

When comparing BPTB to HT, three recent RCTs demonstrated no significant differences between subjective IKDC and Lysholm scores [53, 88, 112]. Additionally, a multicenter RCT with 16-year follow-up revealed no statistical differences in PROs between both graft options [10]. These RCTs have been reinforced by several large registry studies [35, 102, 107, 113], systematic reviews, and meta-analyses [21, 90, 133] showing no difference in PROs between patients treated with BPTB or HT. Similarly, no significant differences have been reported among other mid- to long-term studies using the transtibial approach [14, 34, 46, 112, 130].

The reported results of QT and HT are similar to those of BPTB and HT. In a recent prospective RCT, Lind et al. [71] compared 50 patients treated with QT to 49 patients treated with HT and found no significant differences in PROs. Similarly, no significant differences in PROs were reported in competitive football players [82]. A registry study including 479 patients and two matched-pair analysis further revealed no significant difference between PROs following isolated QT or HT ACL-R in short- and after minimum five years [109–111]. Recent smaller observational studies as well as systematic reviews and metanalyses have confirmed the findings of the above-mentioned comparative studies, showing comparable PROs between patients treated with both graft options [2, 9, 21, 86, 91, 95, 99, 127].

While allografts were historically associated with inferior clinical and patient reported outcomes, recent studies using non-irradiated and non-chemically treated allografts produce comparable patient satisfaction rates and PROs to autografts [11, 24, 36, 59, 128, 135].

### Graft failure rates

Graft failure is multifactorial. Risk factors include male gender [105], younger age [57, 58, 62, 89, 105, 109], family history [17, 137], ethnicity [137], lower body mass index (BMI) [137], increased posterior tibial slope [25, 28, 40, 131], high activity level [17, 57, 58, 109] and concomitant injuries [137]. As many of these factors are non-modifiable, operative technique and graft choice remain easily adjustable factors influencing postoperative outcomes and re-rupture rates [31, 98, 102, 106, 107, 113, 133, 137].

When comparing graft failure rates, care must be taken with terminology, as the terms "graft rupture," “failure rates,” and "revision surgery" are often used interchangeably and interpreted inconsistently. Particularly in registry studies, “revision surgery” may be reported rather than graft ruptures, as determined by postoperative MRI or clinical examination. This may lead to underestimation of true re-rupture rates. In terms of re-rupture, BPTB has long been considered the gold standard, demonstrating decreased rates compared to HT and allograft [3, 35, 65, 74, 76–79, 124, 137]. However, RCTs and observational studies comparing BPTB and QT report similar graft rupture rates, ranging from 1.4—7.5% and 2.0—5.1%, respectively [8, 37, 45, 100]. These results have been supported in recent systematic reviews and meta-analyses showing no significant difference between both graft options [21, 91].

There is extensive evidence on ACL revision surgery rates between BPTB and HT. Out of eleven registry studies, nine reported a significant relationship between revision rate and graft choice, with patients undergoing HT ACL-R having an up to two times higher risk of revision [3, 35, 65, 74, 76–79, 124]. In contrast, four systematic reviews and meta-analyses reported no statistically significant difference in re-rupture and reoperation rates; however, a tendency toward higher re-rupture rates for HT remains [21, 41, 90, 133].

When comparing failure rates of QT to HT, high-level evidence is still lacking. Two RCTs including 99 and 51 patients respectively, found no significant difference between both graft options in the short term [47, 71]. These results are supported by other short-term observational studies in adult [2, 15, 60, 111, 127] and pediatric patients [99]. Contrary to the above-mentioned findings, a recent registry study including 875 patients showed a 2.7 times higher probability of revision surgery when an HT (4.9%) was used compared to QT (2.8%). This difference was even more pronounced in high-level athletes (Tegner activity score ≥ 7), with revision surgery rates of 11.1% and 5.0%, respectively. In less active patients, low revision rates with minor differences were observed (QT: 3.0%, HT: 4.2%). Interestingly, patients with QT showed no difference in the rate of ipsilateral revision surgery and the number of contralateral ACL-R compared to those treated with HT. This indicates a possible superiority of the QT to lower the graft rupture risk to the level of the uninjured, contralateral leg [109]. Similarly, a recent mid-term, matched-pair comparative study revealed no statistically significant difference between both graft options (QT: 17.8%; HT: 22.2%). In highly active patients (Tegner-activity-level ≥ 7), the re-rupture rate increased to 37.5% in the HT group while remaining constant in the QT cohort (22.2%). Results of recent systematic reviews and meta-analyses are inconclusive, reporting either higher [52, 94] or equal [21, 91, 120] re-rupture and revision surgery rates for HT versus QT.

There is extensive but contradicting evidence comparing graft rupture rates between allograft and autograft. Allografts are thought to have higher rupture and reoperation rates, with an up to sixfold increased risk of failure when compared to autograft, especially in young and active patients [18, 58, 63, 72, 96, 126]. Sterilization using radiation, especially with doses greater than 20 kGy, has been implicated as a likely cause due to unfavorable biomechanical effects on the tissue [66, 115].

In more recent studies comparing non-irradiated or fresh frozen allograft to autograft, these higher failure rates have not been consistently reported [11, 24, 26, 68, 135]. Notably, the literature suggests that allografts are now predominantly used in older and less active patients, two well-known factors that lower graft failure rates [26, 85, 103]. This change in indication resulted due to higher graft failure rates observed in young and active individuals with the use of allograft [27, 57, 58, 96, 129]. The Multicenter Orthopaedic Outcomes Network (MOON) registry has shown that changing the indications for allograft based on patient age and sport activity have resulted in a 68% decrease in graft failure rates. However, the odds of failure with allograft in this study remained 9.5 times higher compared to autograft. [58]. Thus, although several systematic reviews and meta-analyses comparing autograft to non-irradiated or fresh frozen allograft have reported no significant differences in failure rates in older patients [24, 134, 136], the use of allograft in young and active individuals remains unacceptably high and is therefore not recommended in this age group [18, 50, 58, 63, 72, 126].

### Strength recovery

Regaining normal extensor and flexor muscle strength after ACL-R, measured by a limb symmetry index (LSI) of > 90%, is a key focus of rehabilitation. The goal is to ensure safe return to sport and work, as inadequate strength has been associated with poorer function, altered biomechanics, and an increased risk of further knee injury [38, 116, 138]. Isokinetic strength testing is considered the “gold-standard” for postoperative strength testing, however varied testing protocols limit the comparability of studies [43]. When comparing different graft options, recent systematic reviews and meta-analyses demonstrate different outcomes [56].

Comparing QT- to BPTB and HT, significantly increased isometric quadriceps weakness at 5–8 months postoperatively with QT, but no significant difference between groups at 9 to 15 months has been demonstrated [49]. Conversely, postoperative hamstring weakness at 5 to 8 months was more pronounced in the HT group compared with the QT group [49]. Other studies have reported similar results, with initial postoperative extensor strength deficits but equal results one year following ACL-R with QT [19, 29]. Isokinetic hamstring:quadriceps ratios are significantly higher for QT compared to HT [82, 117].

When using HT, isokinetic flexor strength is significantly reduced compared to QT, and the deficit may persist for up to two years [19, 29, 70]. Similar data, with no difference in extensor strength but decreased flexor strength when using HT, is also reported when comparing BPTB and HT [6, 42, 67]. Interestingly, a recent study showed that maximal hamstring strength, but not explosive hamstring strength improved over time following ACL-R using HT [114]. Comparing QT to BPTB, similar levels of quadriceps recovery have been observed in the short term [39, 51].

### Return to sport

Return to sport (RTS) following ACL-R is a commonly utilized and clinically important outcome measure. Despite its prevalence, this outcome is often reported in a variety of ways, making it difficult to compare patient subgroups. A meta-analysis found an overall 82% RTS rate following ACL-R, however the rate dropped to 63% when looking at RTS at the same level [5]. Many factors are thought to impact RTS including patient factors such as age, gender, compliance with rehabilitation, and patient confidence, as well as surgical factors such as concomitant injuries and graft choice.

There are few studies in the literature specifically comparing graft choice and its impact on successful RTS, but the consensus appears to find no difference between various graft types. Currently, the literature shows no difference between BPTB and HT in RTS rates. A study focusing on 100 soccer players who underwent ACL-R with either BPTB or HT revealed an overall return to play rate of 72% at 1 year follow up with 85% of those patients returning at the same level or higher [12]. This study highlighted that graft choice did not predict RTS rates [12]. Similarly, a case control study looking at athletes under the age of 25 revealed a non-statistically significant difference in return to preinjury activity level between BPTB patients (57%) and HT patients (43%) [84]. A recent meta-analysis looking at 2,348 athletes had similar findings, with no difference between HT and BPTB in initial return rates (81% and 71%, respectively), as well as no difference between rates of return to preinjury level (50% and 49%, respectively) [23].

In regard to QT, a retrospective study looking at 5-year follow up for 291 young active patients demonstrated a 73% RTS at preinjury level with a mean time of 8 months to return [32]. Although RTS rates for QT appear promising, there are few high-level studies comparing RTS rates with other graft types. A recent randomized controlled trial looking at patients 18 years or older who were randomized to ACL-R with either HT or QT revealed no difference in mean time to RTS at 2-year follow-up [47]. Similarly, a prospective cohort study of 875 patients revealed no difference RTS rates at preinjury level when comparing QT (67%) and HT (74%) [109].

While allograft is an uncommon graft choice in young athletes, the literature frequently reports no difference in RTS rates between autograft and allograft. A recent study compared 78 collegiate level soccer players who underwent ACLR with BPTB (66%), HT (17%), allograft (10%), and QT (1%). The overall mean RTS time was 6 months. There was no difference in RTS rates based on graft selection when comparing all autograft and allograft patients (QT: 100%, BPTB: 90%, HT: 77%, allograft: 75%) [48]. Conversely, a separate study compared 182 collegiate football players who underwent ACL-R with BPTB, HT, or allograft. Overall, 85% of players had autograft and 15% allograft, with the results indicating a significantly higher RTS rate of 85% in autograft compared to 69% in allograft patients [22].

While the current literature highlights that there may be no difference in RTS following ACL-R with various graft types, there is a need for further research on how to improve rates of return to the same level of sport amongst all graft types.

### Complications and donor site morbidity

Surgical techniques continuously evolve not only to improve functional postoperative outcomes, but also to decrease complications and donor site morbidity. Knowledge of the various advantages and disadvantages of each graft option is fundamental to individualized ACL-R. Of course, one of the primary benefits of allograft use is the avoidance of donor site morbidity.

When considering complications and donor site morbidity related to graft choice, it is important to distinguish between minor and major complications. Minor donor site morbidities include persistent anterior knee pain, sensory loss of the lower leg, donor-site tendinopathy, scarring, cosmetic issues, and discomfort during kneeling (in patients without daily kneeling activities). Major complications besides graft rupture and contralateral ACL rupture include kneeling pain in patients who kneel during daily living, patellar fracture, extensor tendon rupture, and infection.

Anterior knee and kneeling pain is the most common postoperative complication related to graft choice, reported in up to 21.5% of patients [1]. Evidence suggests that patients treated with BPTB have a significantly higher incidence (up to 72%) of postoperative anterior knee and kneeling pain compared to those treated with HT (up to 44%) or QT (up to 9.3%), possibly attributable to injury of the infrapatellar nerve and/or irritating of the Hoffa fat pad during BPTB harvest [10, 33, 41, 81, 92, 104, 110, 111, 118, 125]. When comparing HT to QT, no significant differences [2, 92, 119, 127] or slightly better outcomes were reported for QT [71, 110]. These favorable outcomes for QT over HT were supported by a recent metanalysis [52].

While minor donor site morbidities are irritating, severe complications like patellar fracture or extensor tendon rupture have a major impact on a patient's life and recovery. Patella fracture after ACL-R with autograft using bone blocks ranges between 0.1% and 2% [39, 45, 61, 123], but may be as high as 8.8% when including occult fractures [30]. Recently safe zones for bone block harvest have been described. A precise surgical technique is recommended, with harvest localization medial to midline and without exceeding 50% of the patellar thickness and patellar height [30, 93]. Compared to patella fractures, ruptures of the quadriceps or patella tendon after ACL-R are even rarer 1% and mainly reported only as case reports [69, 83, 87, 118].

Superficial and deep surgical site infection (SSI) after ACL-R is a rare but major complication, with an incidence between 0.32% and 1.1% [64, 75, 80]. Recently, evidence has emerged showing graft choice has an influence on the rate of postoperative SSI [64, 75, 80]. An up to eight times higher risk of SSI was reported in patients treated with HT compared to those with BPTB [75]. These findings have been confirmed by a recent large, single-center study showing that HT and allograft are associated with a five times higher risk of postoperative infection compared to BPTB [80]. When comparing all four graft options, QT seems to have the lowest rate of infection. The reason for differing rates of SSI with different graft options remains unclear, however contamination after harvest or preparation has been observed in up to 59.4% of cases and is the most accepted hypothesis [101, 132].

Compared to autografts, allografts have the advantage of reduced surgical time, lower donor site morbidity, and more predictable graft size but are believed to have a higher infection rate compared to autografts [20, 50]. Although rare, there is a risk of contamination of the implanted allograft and pathogens are often highly virulent, such as Clostridium or other bowel microorganisms [50].

### Authors’ choice

With all graft options having advantages and drawbacks (Table 1), graft choice must be individualized and chosen in accordance with the patient. For primary ACL-R in adults, the authors prefer QT or allograft. For younger and active patients, the authors prefer QT-A because of its favorable biomechanical characteristics, predictable size, and faster incorporation compared to allograft (for details see “Current Trends In Graft Choice For Primary Anterior Cruciate Ligament Reconstruction—Part 1”). QT also demonstrates lower donor site morbidity compared to BPTB-A and a tendency towards lower graft re-rupture rates compared to HT, especially in highly active patients. Particularly in young and high-level athletes, the authors do not recommend the use of allograft, mainly due to the slower graft incorporation process which may result in excessive mechanical graft stress and higher failure rates when paired with the desire to quickly return to sport. In contrast, in older and less active patients, allograft is preferred due to shorter surgical times, lower donor site morbidity, and comparable PROs compared to autograft.

**Table 1:** Advantages, disadvantages, and the optimal patient for different ACL graft options

Graft Type	Optimal Patient	Advantages	Disadvantages
QT	< 35 years old High-level pivoting sport and/or high physical demand Work, activity or sport that requires kneeling Skeletally immature patients	Comparable graft rupture rates to BPTB Lower donor site morbidity than BPTB but comparable to HT Possibility of single side bone-block harvest Possibility of individualized graft size by harvesting partial- or full thickness graft Less flexion strength loss compared to HT	No long-term outcomes Decreased extensor strength Risk of patellar fracture or quadriceps tendon rupture
BPTB	< 35 years old High-level pivoting sports high physical demand	Bone-to-bone healing and therefore possibly more aggressive rehabilitation Low graft rupture rates comparable to QT High return to sport rates	Highest rate of donor site morbidity and anterior knee pain Higher rates of OA progression Risk of patellar fracture or patella tendon rupture No option for skeletally immature patients Possible higher risk of contralateral ACL rupture Decreased extensor strength
HT	Moderate sport and/or activity level Small ACL footprint Work, activity or sport that requires kneeling Skeletally immature patients	Lower donor site morbidity compared to BPTB Possibility of individualized graft size by additional gracilis tendon harvest and different graft configurations No risk for patellar fracture or extensor mechanism rupture Lower OA progression than BPTB	Higher graft rupture rates compared to QT and BPTB, especially in young and active patients Increased ATT after HT ACL-R, possibly due to reduction in hamstring force Tendency towards higher surgical site infection rates Decreased flexion strength
Allograft	> 40 years old Low activity level and/or physicaldemand Multiligament Knee Injury	No donor site morbidity Faster operation time More predictable graft size	Higher graft rupture rates compared to QT and BPTB, especially in young and active patients Slower rehabilitation speed due to delayed graft maturation and incorporation Increased costs

*QT* Quadriceps Tendon Autograft, *BPTB* Bone-Patellar-Tendon-Bone Autograft, *HT* Hamstring Tendon Autograft, *ACL-R* Anterior Cruciate Ligament Reconstruction, *ATT* Anterior Tibial Translation, *OA* Osteoarthritis

## Conclusion

Graft choice affects postoperative outcomes after ACL-R and normal knee kinematics is not fully restored after surgery. Patients with hamstring tendon autograft may experience an increase in ATT and a decrease in flexion strength compared to those treated with BPTB or QT. Contrary, extensor strength is affected in patients with BPTB and QT. While patient reported outcomes are not influenced by graft choice, evidence suggests favorable postoperative graft rupture rates in patients treated with BPTB and QT autografts over HT or allografts. With regards to return to sports the consensus appears to find no difference between various graft types. Postoperative donor site morbidity is highest in BPTB, comparable between HT and QT and absent in allografts. With all graft options having advantages and drawbacks, graft choice must be individualized and chosen in accordance with the patient.
